# Managing your life as an IR — perspective of a young IR

**DOI:** 10.1186/s42155-026-00705-8

**Published:** 2026-05-16

**Authors:** Lora Grbanović, Ana Marija Alduk

**Affiliations:** Clinical Department of Diagnostic and Interventional Radiology, University Hospital Centre Zagreb, Zagreb, Croatia

Interventional radiology (IR) is a specialty defined by innovation, technical excellence, and the ability to make a tangible difference in patients’ lives. For many of us, these qualities are what first attract us to the field and continue to fuel our motivation; however, behind these sources of professional fulfillment lies a demanding practice reality: a high-stress, fast-paced environment, complex procedures with critical outcomes and the emotional toll of patient care. This article is written from the perspective of a young interventional radiologist, here referring to IR trainees, including residents and fellows.

Reported burnout rates in IR are among the highest across medical specialties, with studies suggesting rates of up to 72% among all practitioners [1] and 88% among IR residents [2]. This is unsurprising given the steep learning curve trainees face in procedural skills and clinical decision-making. IR is a rapidly evolving field that demands continuous learning and adaptation. The high stakes of the specialty translate into equally high professional demands, requiring dedication, attention to detail, and consistently high performance. These pressures can contribute to the development of imposter syndrome, which is already common among physicians [3].

Identifying stressors and developing strategies to manage them are essential for long-term sustainability in the field. A high volume of procedures, long hours in the angiography suite, and on-call responsibilities often challenge healthy professional boundaries, allowing work to encroach on personal life and relationships. Concerns about radiation exposure, particularly for women of childbearing age, can further increase work-related stress. Current evidence is reassuring: with appropriate protection measures, fetal exposure is extremely unlikely to reach harmful thresholds, and offspring of medical radiation workers have not shown convincing evidence of increased cancer risk [4]. The psychosocial impact of procedural complications on interventional radiologists is important, but frequently underrecognized. “Second Victim Syndrome” refers to the emotional consequences of adverse events, which can include anxiety, depression and shame in healthcare providers [5]. Building supportive team environments may help mitigate this impact.

The main organizational challenge in IR is an unpredictable workflow. Planned schedules are frequently disrupted by urgent cases, requiring constant adaptation and reprioritization. Time management is essential to maintain efficiency and preserve capacity for responsibilities beyond immediate clinical tasks. One helpful framework for task prioritization is the Eisenhower Matrix, which categorizes tasks according to urgency and importance [6] (Fig. 1).

**Fig. 1:**
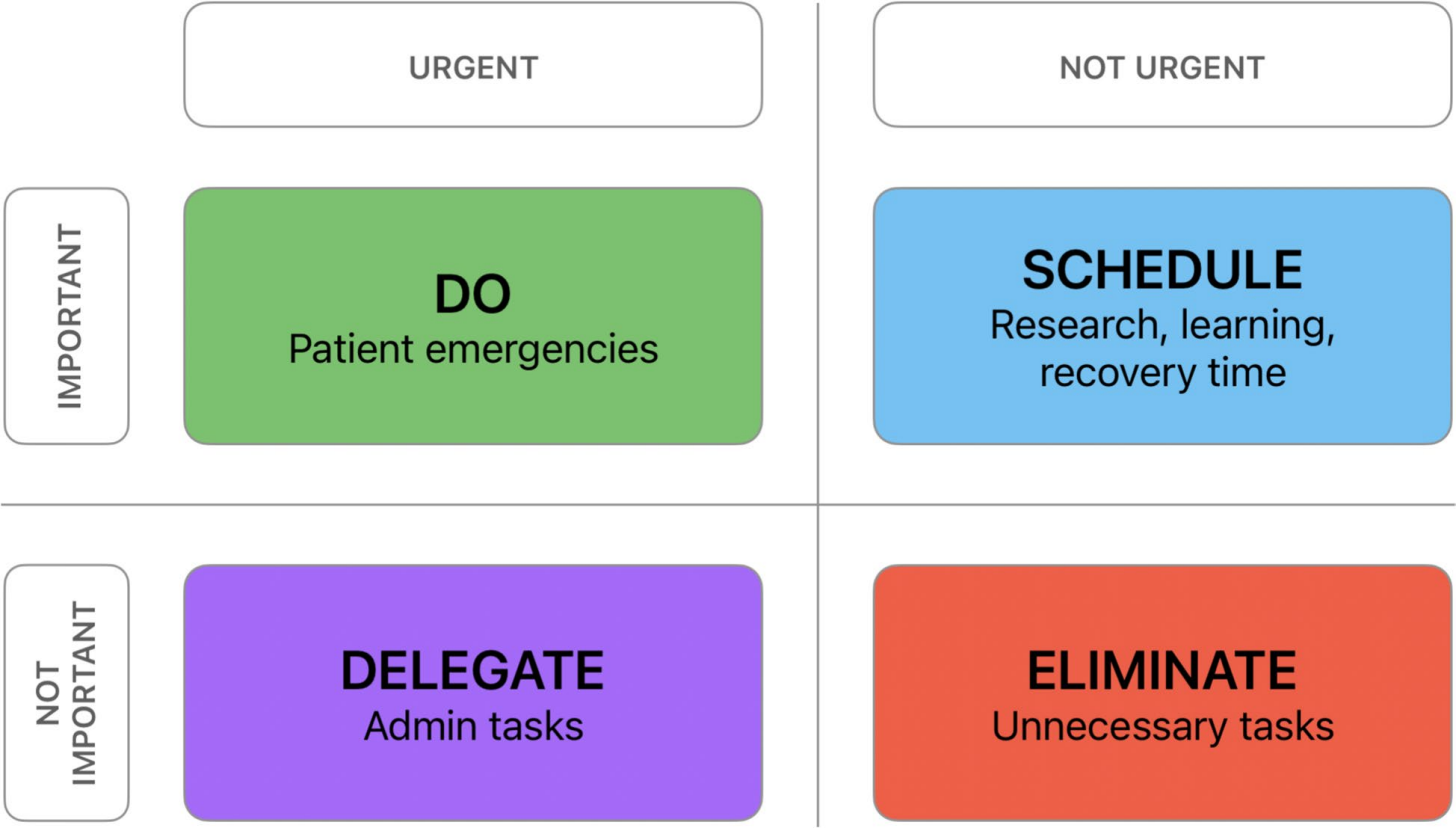
Eisenhower Matrix applied to interventional radiology tasks

In IR, truly urgent and important tasks, such as emergent procedures, naturally require immediate attention. The greater difficulty comes from managing tasks that feel urgent but have limited long-term impact. Administrative and low-value tasks may include non-essential documentation and meetings, while unnecessary tasks refer to activities with limited clinical, educational, or professional value. These can easily displace activities that are important but not urgent, such as learning, research, career planning or time for recovery. Given the unpredictability of IR practice, the goal is not strict schedule control but the deliberate allocation of time to activities that support professional development and personal well-being.

Effective communication is both a clinical necessity and a practical time-management tool. Clear communication with other members of the IR team, referring physicians, patients and their families helps prevent schedule disruptions and keeps workflow running smoothly. It also reinforces professional boundaries: by clearly articulating their expectations and limitations to the clinical team and departmental leadership, young interventional radiologists can contribute to setting limits on work hours, on-call duties and administrative responsibilities. This includes the ability to decline additional responsibilities that fall outside one’s current scope and priorities. In practice, the ability to set such limits is often shaped by departmental culture and institutional support.

Building strong support systems is fundamental for professional sustainability. Senior mentors and peer support networks can provide guidance in clinical practice and career development, while a constructive departmental culture can support relationship building, open feedback, and reducing daily strain through collaboration and realistic workload expectations. Mental health interventions, such as resilience-building, physical activity and psychoeducation, have been shown to improve well-being among healthcare workers [7]. Attention to physical well-being, including adequate rest and procedural ergonomics, is equally essential for long-term sustainability. Personal support outside the hospital, including family, partners, and friends plays a vital role in maintaining perspective and emotional balance. Together, these professional and personal support systems help young interventional radiologists navigate demanding periods without becoming isolated.

In conclusion, a career in interventional radiology is a marathon, not a sprint, requiring ongoing commitment to learning, personal growth, and professional development. Sustaining a career depends on intentional choices: prioritizing self-care, cultivating strong support systems, setting boundaries, and maintaining a passion for the specialty while being aware that not all goals can be pursued simultaneously. Ultimately, the goal is not a flawless practice but the consistent pursuit of excellence alongside a balanced life, enabling long-term fulfillment in this challenging yet rewarding field.

## Data Availability

Not applicable.

## Abbreviation

IR: Interventional radiology

## References

[1] Bundy JJ, Hage AN, Srinivasa RN, Gemmete JJ, Lee E, Gross JS, et al. Burnout among interventional radiologists. J Vasc Interv Radiol. 2020;31(4):607-613.e1. 10.1016/j.jvir.2019.06.002. 31345730 10.1016/j.jvir.2019.06.002

[2] Lima N, Davidson K, Bhatnagar V, Khodorov G, Bryant H, Hoffer E. Abstract No. 357: assessing burnout amongst interventional radiology trainees. J Vasc Interv Radiol. 2025;36(3 Suppl):S161–2. 10.1016/j.jvir.2024.12.421.

[3] Beckman TJ. The imposter syndrome in physicians. Mayo Clin Proc. 2022;97(11):1964–5. 10.1016/j.mayocp.2022.09.014. 36333010 10.1016/j.mayocp.2022.09.014

[4] Ozcan BB, Kunzelman Anderson J, Nadim B, Sajan A, Fung KFK. Understanding radiation safety: essential knowledge for radiology residents. Radiographics. 2026;46(3):e250028. 10.1148/rg.250028. 41678366 10.1148/rg.250028

[5] Oseni AO, Chun JY, Morgan R, Ratnam L. Dealing with complications in interventional radiology. CVIR Endovasc. 2024;7(1):32. 10.1186/s42155-024-00442-w. 38512496 10.1186/s42155-024-00442-w PMC10957835

[6] Kennedy DR, Porter AL. The illusion of urgency. Am J Pharm Educ. 2022;86(7):8914. 10.5688/ajpe8914. 34716138 10.5688/ajpe8914 PMC10159458

[7] Mahanjana SK, Pitso LA, Ncube MV. Mapping intervention strategies and mental health support journeys in addressing mental health challenges among healthcare professionals - a scoping review. BMC Psychol. 2025;13(1):651. 10.1186/s40359-025-02981-w. 40598618 10.1186/s40359-025-02981-w PMC12210555

